# Supplementary material for: FKBP4 regulates 5-fluorouracil sensitivity in colon cancer by controlling mitochondrial respiration
Source: Life Sci Alliance. 2022 Aug 18;5(11):e202201413. doi: 10.26508/lsa.202201413 (PMC9389594; doi:10.26508/lsa.202201413)
Supplement: Supplementary file 1 [file LSA-2022-01413_TableS1.docx]

**Supplementary Table S1**

Detailed information regarding the antibodies, siRNAs, plasmids, and chemicals used in this study.

| **Antibodies** | | | |
| --- | --- | --- | --- |
| Name | Source | Cat. No. | |
| anti-FKBP4 antibody | Cell Signaling Technology | 11826 | |
|  |  | Dilutions: 1:1000 (WB) | |
| anti-α-Tubulin antibody | Cell Signaling Technology | 2144 | |
|  |  | Dilutions: 1:1000 (WB) | |
| anti-Lamin B1 antibody | Cell Signaling Technology | 12586 | |
|  |  | Dilutions: 1:1000 (WB) | |
| anti-Tom20 antibody | Santa Cruz Biotechnology | sc-17764 | |
|  |  | Dilutions: 1:1000 (WB) | |
| anti-CYCS antibody | Abcam | ab13575 | |
|  |  | Dilutions: 1:1000 (WB) | |
| anti-TFAM antibody | Cell Signaling Technology | 7495 | |
|  |  | Dilutions: 1:1000 (WB) | |
| anti-6xHis tag antibody | Abcam | ab18184 | |
|  |  | Dilutions: 1:1000 (WB) | |
| anti-COA6 antibody | Novus Biologicals | NBP1-85948 | |
|  |  | Dilutions: 1:500 (WB) | |
| anti-SCO1 antibody | Sigma-Aldrich | HPA021565 | |
|  |  | Dilutions: 1:1500 (WB) | |
| anti-SCO2 antibody | Invitrogen | PA5-76209 | |
|  |  | Dilutions: 1:2000 (WB) | |
| anti-MT-CO1 antibody | Cell Signaling Technology | 62101 | |
|  |  | Dilutions: 1:1000 (WB) | |
| anti-MT-CO2 antibody | Cell Signaling Technology | 31219 | |
|  |  | Dilutions: 1:1000 (WB) | |
| anti-MT-CO3 antibody | Sigma-Aldrich | SAB1305244 | |
|  |  | Dilutions: 1:1000 (WB) | |
| **siRNA sequences (5’-3’)** |  |  | |
| siCtrl_1: CGCUAUGAGUUGUGUGUGUUU and AACAAACUCACCACAACUCAUAGCG | | | |
| siCtrl_2: CCGGGAGUUGUGUCUAUGUGUCCUU and AAGGACACAUAGACACAACUCCCGG | | | |
| siFKBP4_1: CGCUUGUAUUUGGGUGUGUU and AACAACUCCACCUCAAAUACAAGCG | | | |
| siFKBP4_2 (3’ UTR): CCGCUGGGAUUUGUGUCUAUGGCUU and AAGCCAUAGACACAAAUCCCAGCGG | | | |
| siCOA6_1: CAAUGCAAGAAGUUAAGAAGCUCUU and AAGAGCUUCUUAACUUCUUGCAUUG | | | |
| siCOA6_2 (3’UTR): GAUCAAGAACCAGAAGAUCUGUGAA and UUCACAGAUCUUCUGGUUCUUGAUC | | | |
| **Plasmids** | | | |
| pLenti6-MTS1-FKBP4-6xHis | | | |
| pLenti6-MTS2-FKBP4-6xHis | | | |
| pLenti6-NLS-FKBP4-6xHis  pLenti6-COA6 | | | |
| **Chemicals** | | | |
| KCN (Potassium cyanide) | | | Sigma-Aldrich |
| ADDA5 (ADDA 5 hydrochloride) | | | Sigma-Aldrich |
